# Prevalence, Virulence Genes, Drug Resistance and Genetic Evolution of *Trueperella pyogenes* in Small Ruminants in Western China

**DOI:** 10.3390/ani14202964

**Published:** 2024-10-14

**Authors:** Yuchen Wei, Bin Wang, Ke Wu, Chenxiao Wang, Xindong Bai, Juan Wang, Zengqi Yang

**Affiliations:** 1 College of Veterinary Medicine, Northwest A&F University, Yangling 712100, China; 2 College of Life Science, Sichuan University, Chengdu 610064, China; 3 Key Laboratory of Prevention and Control of Major Ruminant Diseases, Ministry of Agriculture and Rural Affairs (Western Region), Northwest A&F University, Yangling 712100, China; 4 Engineering Research Center of Novel Animal Efficient Vaccines of the Ministry of Education, Northwest A&F University, Yangling 712100, China

**Keywords:** *Trueperella pyogenes*, goats, sheep, virulence genes, antimicrobial resistance, whole genome sequencing

## Abstract

**Simple Summary:**

*Trueperella pyogenes* is a significant opportunistic pathogen that can infect a wide range of animals and humans. Understanding its virulence factors and antibiotic resistance mechanisms is essential for the prevention and control of infections. Presently, research predominantly concentrates on *Trueperella pyogenes* in dairy cows and pigs, with limited investigations into its prevalence in small ruminants like sheep and goats, despite their importance as food sources. In light of the rapid expansion of China’s sheep and goat industry, this study focused on the isolation rate of *Trueperella pyogenes* from clinical samples obtained from various farms in western China. The objectives were to identify virulence genes and assess drug resistance characteristics in the isolated strains. Furthermore, the genetic relationships and virulence gene profiles, including antibiotic resistance gene carriage, were evaluated in selected isolates. The study underscored the threat posed by *Trueperella pyogenes* to sheep and goats and highlighted the high levels of antibiotic resistance observed. These findings provide crucial insights for the prevention and treatment strategies on farms and advocate for the prudent use of antibiotics to mitigate the transmission of drug-resistant bacteria through the food chain, thereby protecting human health.

**Abstract:**

*Trueperella pyogenes* is a significant opportunistic pathogen that causes substantial economic losses in animal agriculture due to its ability to infect various animal tissues and organs. Limited research has been conducted on the prevalence and biological characteristics of *T. pyogenes* isolated from sheep and goats. This study aimed to isolate *T. pyogenes* from clinical samples of sheep and goats in western China, examining genetic evolutionary relationships, antibiotic resistance, and virulence genes. Between 2021 and 2023, standard bacteriological methods were used to isolate and identify *T. pyogenes* from 316 samples (209 from goats and 107 from sheep) collected from 39 farms. Susceptibility to 14 antibiotics was tested using broth microdilution per CLSI guidelines, and PCR detected eight virulence genes. Whole-genome sequencing analyzed genetic relationships and gene carriage status in 39 isolates. The results indicated that 86 strains of *T. pyogenes* were isolated from 316 samples, yielding an isolation rate of 27.2% (goats n = 47, 22.5%; sheep n = 39, 36.4%). The virulence genes *plo*, *cbpA*, *nanH*, *nanP*, *fimA*, *fimC*, and *fimE* were present in 100%, 66.7%, 64.1%, 71.8%, 69.2%, 59.0%, and 82.1% of isolates, respectively, with none carrying the *fimG* gene. The dominant virulence genotype was *plo*/*nanH*/*nanP*/*fimA*/*fimC*/*fimE*. The isolates exhibited resistance to erythromycin (44.2%, 38/86), gentamicin (38.4%, 33/86), sulfamethoxazole/trimethoprim (37.2%, 32/86), tetracycline (32.6%, 28/86), and streptomycin (32.6%, 28/86), and low resistance to chloramphenicol (14.0%, 12/86), ciprofloxacin (7.0%, 6/86), penicillin (5.8%, 5/86), and clindamycin (4.7%, 4/86). All isolates were susceptible to cefotaxime, vancomycin, and linezolid. Among the 86 isolates, 37 (43.0%) displayed multidrug resistance (MDR) characteristics. The whole genome sequencing of 39 isolates identified eight types of resistance genes, including *ant(2″)-Ia*, *ant(3″)-Ia*, *cmlA1*, *cmx*, *erm(X)*, *lnu(A)*, *sul1*, and *tet(W)*. Except for *tet(W)*, *erm(X)*, and *sul1*, the other resistance genes were reported for the first time in *T. pyogenes* isolated in China. The drug susceptibility test results and resistance gene detection for the isolated strains were consistent for tetracycline, erythromycin, gentamicin, and sulfisoxazole. Similar allelic profiles and genetic evolutionary relationships were found among isolates from different farms. This study highlights the antibiotic resistance status and virulence gene-carrying rate of Trueperella pyogenes, providing a basis for clinical medication.

## 1. Introduction

*Trueperella pyogenes* is a Gram-positive, short coryneform bacterium that commonly inhabits the skin and mucous membranes of the respiratory, gastrointestinal, and genitourinary tracts of animals [1]. Being a significant opportunistic pathogen, it can infect a range of animals, including companion, wild, and domestic animals, with purulent infections [2], leading to diseases such as metritis [3], mastitis [4], pneumonia [5], abscesses [6], and so on. It generates significant financial losses and has a significant effect on the livestock business. The majority of *T. pyogenes* that are being researched now come from food animals such as pigs, sheep, goats, and cattle [7], and there are also reports of human infection with *T. pyogenes* [8,9]. This emphasizes the potential for transmission of this harmful microorganism from animals to humans, posing a significant risk to both food safety and public health.

Virulence factors are crucial in determining bacterial pathogenicity. The putative virulence factors associated with the pathogenicity of *T. pyogenes* include hemolysin (*Plo*), collagen-binding protein (*cbpA*), neuraminidase (*nanH*, *nanP*), and fimbriae proteins (*fimA*, *fimC*, *fimE*, *fimG*). In general, the spectrum of genes encoding particular virulence factors is independent of infection type and host species [10]. Nonetheless, certain situations indicate a correlation between specific virulence genes and particular forms of infection [11]. The interactions between pathogens and hosts in *T. pyogenes* infection are not well understood., and it is uncertain how virulence factors contribute to the development of infection. Therefore, researching and monitoring virulence genes, as well as studying infection and carriage, can provide more information about the virulence genotype and pathogenic effects of the pathogen. This can help to identify suitable targets for effective prevention and control of the pathogen.

Antibiotics such as aminoglycosides, β-lactams, macrolides, and sulfonamides are commonly used in veterinary medicine to treat bacterial infections, including those caused by *T. pyogenes* [1,12,13]. More and more studies have shown that the antibiotic resistance of *T. pyogenes* has become a serious problem. This not only brings great difficulties to clinical treatment but also poses a serious threat to public health security. There has been extensive research on drug resistance phenotypes in strains derived from livestock and wild animals like cows [14], pigs [15], European bison [16], and others. However, there is limited data on the drug resistance phenotypes and resistance genes of strains in Chinese sheep and goats. The sheep and goat farming industry in China is rapidly developing, but increased stocking density and frequent sheep transportation have led to a decline in overall immunity levels. It is crucial to determine the clinical isolation rate of this pathogen for further risk assessment. Whole-genome sequencing analysis can provide insights into genetic evolutionary relationships, pathogenicity, antibiotic resistance, and epidemiological characteristics.

In summary, it is important to monitor the virulence and drug resistance of *T. pyogenes* isolated from samples derived from different diseased tissues of sheep in order to control and reduce its harm to the sheep industry and public health. This study aims to investigate the isolation rate, virulence gene carriage status, and drug resistance characteristics of *T. pyogenes* derived from different diseased tissues of sheep in parts of northwestern China. Additionally, whole-genome sequencing technology is being used to study the genomic characteristics of strains from different sources, to understand the carriage status of virulence genes and drug-resistant genes at the genetic level, and to compare them with antibiotic resistance phenotypes. This study’s findings enhance our understanding of the isolation prevalence, virulence gene carriage, and antibiotic resistance profiles of *T. pyogenes* strains isolated from sheep and goats. The genomic data generated will serve as a foundational resource for future research into the pathogenic mechanisms of *T. pyogenes* and for the development of clinical treatments within the context of sheep and goat husbandry.

## 2. Materials and Methods

### 2.1. Biosafety Statement

For the clinical isolates of *T. pyogenes*, we strictly adhered to the regulations of the State Council of the People’s Republic of China and its guidelines on the biosafety of pathogen microbiology laboratories (000014349/2004-00195). We implemented all necessary safety procedures to prevent the transmission and infection of pathogens.

### 2.2. Material Collection

Between 2021 and 2023, a total of 316 clinical specimens were collected from sheep (*n* = 107) and goats (*n* = 209) on 39 farms in Shaanxi Province, Gansu Province, and Tibet, China. The specimens were classified into 5 categories based on clinical manifestations: pneumonia (*n* = 42), subcutaneous abscess (*n* = 121), mastitis (*n* = 57), ulcerated skin (*n* = 37), and metritis (*n* = 59). The sources and quantities of various samples are shown in Table 1. Sample collection details are described below: Pneumonia: Lung samples were collected from deceased sheep and goats exhibiting symptoms of pneumonia during necropsy; Metritis: For sheep and goats with clinical symptoms of metritis, the external genitalia were disinfected, and a long cotton swab moistened with sterile saline was gently inserted into the vagina. The swab was rotated, and the mucous membrane of the uterine cavity was wiped quickly. It was then removed and immediately placed into a sterile centrifuge tube. Subcutaneous abscess: For the sheep observed with subcutaneous abscesses during the survey, the surface of the abscess was disinfected. A disposable sterile syringe was then inserted into the abscess cavity along the central part or base, and about 5 mL of purulent content was collected. Mastitis: After cleaning and disinfecting the surface of the udder of dairy goats with mastitis, 5–10 mL of milk was collected aseptically and divided into sterile centrifuge tubes. Ulcerated skin: Sterile forceps were used to collect scab tissue with fresh ulcerative lesions from each affected sheep, placed in centrifuge tubes containing sterile saline that had been sterilized by autoclaving and properly labeled. These specimens were collected aseptically, placed at 4 °C, and quickly transported back to the laboratory within 24 h for isolation of *T. pyogenes*.

### 2.3. Isolation and Identification

The samples were placed on brain heart infusion (BHI) agar with 5% sheep blood and then incubated at 37 °C in a 5% CO_2_ environment for 48 h. Single colonies showing typical *T. pyogenes* growth were selected for further identification. Bacterial morphology, Gram staining, and biochemical tests were used to identify the species. Use the primers (Forward: 5′-CCAGCAGCCCGGCGGGTAATACG-3′, Reverse: 5′-ATCGGYTACCTTGTACGACTTC-3′) to conduct PCR on the 16S rRNA gene sequence [17]. Send the PCR products for sequencing and comparison to identify the species. Pure cultures of *T. pyogenes* on brain heart infusion (BHI) agar were suspended in tryptic soy broth (TSB) containing 20% glycerol (*v*/*v*) and stored at −20 °C for future use.

### 2.4. Genomic DNA Isolation

Genomic DNA was extracted from overnight cultures of *T. pyogenes* strains in Brain Heart Infusion (BHI) medium and then incubated for 24 h at 37 °C in 5% CO_2_ with shaking at approximately 160 rpm. The genomic DNA of *T. pyogenes* isolates was extracted using the TIANamp Bacteria DNA Kit (Tiangen, Beijing, China), and the quantity and purity of the genomic DNA were determined using a spectrophotometer NanoDrop 1000 (Thermo Fisher Scientific, Waltham, MA, USA). The genomic DNA samples were stored at −20 °C until used.

### 2.5. Virulence Gene Testing

Use the DNA extracted in Section 2.3 as a template for the PCR, and follow the specific primers, PCR reaction system, and conditions outlined in the literature [11], and carry out the eight known putative virulence genes of *plo*, *cbpA*, *nanH*, *nanP*, *fimA*, *fimC*, *fimE*, and *fimG*. The situation was detected, and the PCR reaction products were detected by gel electrophoresis. *T. pyogenes* ATCC19411 was used as the quality control strain.

### 2.6. Determination of Minimum Inhibitory Concentration (MIC)

The minimum inhibitory concentration (MIC) of 14 antimicrobial agents was determined using the broth microdilution method following the recommendations of Clinical and Laboratory Standards Institute M45 [18] and VET 06 [19]. The antibacterial drugs tested included Penicillin, amoxicillin and clavulanate potassium, erythromycin, clindamycin, ciprofloxacin, cefotaxime, vancomycin, sulfisoxazole, tetracycline, florfenicol, streptomycin, chloramphenicol gentamicin, linezolid. The method involved suspending the colony in 0.9% NaCl, adjusting its density to 0.5 McFarland value equivalent to 10^8^ CFU/mL, taking a total of 10 μL of suspension, mixing it with 11 mL of lysed horse blood (LHB) cation-adjusted Mueller–Hinton broth (CAMHB) containing 5% (*v*/*v*), adding 50 μL to each well, and then incubating at 37 °C and 5% CO_2_ for 24 h.

The minimum inhibitory concentration (MIC) is the lowest concentration of an antimicrobial agent that stops the visible growth of bacteria. We also determined the MIC_50_ and MIC_90_ for each antimicrobial agent. We based our selection of resistance breakpoints on the guidelines set by the CLSI document M45 and VET06 *Corynebacterium* genus (including *T. pyogenes*) breakpoints for these antibiotics. The following breakpoints were utilized: penicillin (≥4 g/mL), gentamicin (≥16 μg/mL), erythromycin (≥2 μg/mL), ciprofloxacin (≥4 μg/mL), tetracycline (≥16 μg/mL), clindamycin (≥4 μg/mL), and sulfisoxazole (≥4/76 μg/mL). CLSI M45 provides susceptibility breakpoints for linezolid (≤2 μg/mL) and vancomycin (≤2 μg/mL) but does not specify resistance determination criteria. MICs for streptomycin (≥4 μg/mL) and chloramphenicol (≥8 μg/mL) are interpreted using resistance breakpoints from previously published data [20,21]. *T. pyogenes* ATCC19411 was used as the quality control strain.

### 2.7. WGS-Based Genetic Characterization

To understand the genetic diversity and evolutionary relationship of *T. pyogenes* isolates in this study, 39 strains with different drug susceptibility profiles collected from 16 sheep and 23 goats underwent whole-genome sequencing. The genomic DNA of the 39 isolates was extracted using the TIANamp bacterial DNA kit. Beijing Biomic Biotechnology Co., Ltd. (Beijing, China) utilized Illumina HiSeq to generate 2x150bp paired-end read data, which was then assembled using SPAdes 3.9.0. The complete genome sequences of the 39 strains were deposited in the NCBI GenBank database with the project number PRJNA1091699. Drug resistance gene comparison and analysis were conducted using ResFinder in the CGE online server (http://genepi.food.dtu.dk/resfinder (accessed on 25 May 2024). A BLASTN search and comparison was performed using the accession number and reference sequence reference [22] from the literature to compare the following genes: *plo* (GenBank accession number: AB027461), *cbpA* (GenBank accession number: AY223543.1), *nanH* (GenBank accession number: AF298154.1), *nanP* (GenBank accession number: AY045771.1), *fimA* (GenBank accession number: NZ_CP007519.1), *fimC* (GenBank accession number: NZ_CP007519.1), *fimE* (GenBank accession number: NZ_CP007519.1), and *fimG* (GenBank accession number: NZ_CP007519.1). A phylogenetic tree based on single nucleotide polymorphisms (SNPs) was constructed using CSIPhylogeny (https://cge.food.dtu.dk/services/CSIPhylogeny/ (accessed on 25 May 2024), and iTOL was used to visualize the results.

### 2.8. Statistical Analysis

The statistical analysis was conducted using SPSS (IBM Corporation version 25.0, SPSS Inc., New York, NY, USA), Microsoft Excel 2010 (Microsoft Corporation, Redmond, DC, USA), and GraphPad Prism 9 (Scientific 2D Graphing and Statistics Software, California Inc., San Jose, CA, USA). To check the normality of the MIC distribution, the Kolmogorov–Smirnov test was carried out (*p* < 0.05). Subsequently, comparisons were made using the Mann–Whitney U non-parametric test with relevant corrections for multiple comparisons (*p* < 0.05). A chi-square test was performed to investigate the potential relationship between animal species and MIC distribution and virulence gene-carrying status. A difference was considered statistically significant when *p* < 0.05.

## 3. Results

### 3.1. Prevalence of T. pyogenes Infections in Goats and Sheep in Western China

**Figure 1:**
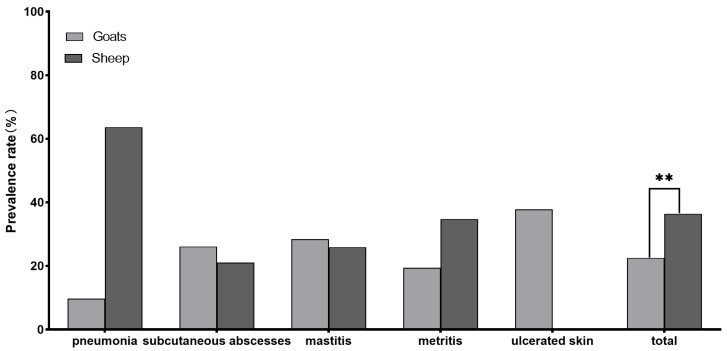
Prevalence rate of *T. pyogenes* for different clinical manifestations in sheep and goats (** *p* < 0.01).

A total of 86 strains of *T. pyogenes* were isolated from 316 samples: 47 from goats and 39 from sheep, with an isolation rate of 27.2% (86/316). The infection rates were 22.5% (47/209) for goats and 36.4% (39/107) for sheep. The breakdown of different tissue sample sources is shown in Table 2. It is important to note that there is no significant difference in the isolation rate of *T. pyogenes* from sheep and goats across different clinical samples. However, as shown in Figure 1, the overall isolation rate of *T. pyogenes* from sheep (36.4%, 39/107) is higher than the isolation rate from goats (22.5%, 86/316), and this difference is statistically significant (*p* = 0.008).

### 3.2. Detection of Virulence Genes

**Figure 2:**
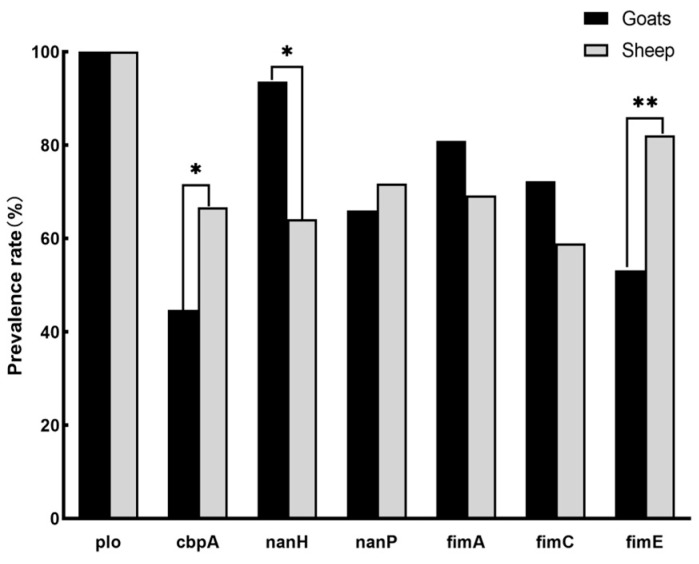
Different virulence gene prevalence rates of *T. pyogenes* isolates derived from sheep and goats (* *p* < 0.05, ** *p* < 0.01).

Figure 2 depicts the transfer of virulence genes in goats and sheep. The *plo* gene was present in all strains (100%, 86/86), while the *fimG* gene was absent. The carrying rate of the *cbpA* gene was 54.7% (47/86), and the carrying rate of *nanH* and *nanP* genes was 80.2% (69/86) and 59.3% (51/86), respectively. The carrying rates of *fimA*, *fimC*, and *fimE* encoding fimbrial-related proteins. The gene-carrying rates were 79.1% (65/86), 66.3% (57/86), and 66.3% (57/86), respectively. At the same time, we found that in some cases, there was a statistical difference between the carrying status of virulence genes and their isolation sources. Correlation: The carrying rate of *cbpA* genes in sheep is 44.7% (21/47), which is significantly higher than that in goats, 66.7% (26/39), *p* = 0.041. The carrying rate of the *nanH* gene in goats was 93.7% (44/47), which was significantly higher than that in sheep at 64.1% (25/39), *p* = 0.013. The carrying rate of *fimE* gene in sheep was 82.1% (32/39), which was significantly higher than that in goats, 53.2% (25/47), *p* = 0.005. Although the carrying rates of *nanP*, *fimA*, and *fimC* genes are different, there is no statistical difference.

Statistical analysis of isolates from various clinical manifestations revealed that the carrying rates of the *fimA* gene in *T. pyogenes* isolates from ulcerated skin and subcutaneous abscess were 92.9% and 81.8%, respectively, which were significantly higher than those isolated from pneumonia(Table 3). The strain exhibits a 30.0% *fimA* gene-carrying rate.

Twenty-five distinct pathogenicity genotypes were discovered (Table S1). Sixteen isolates (18.6%) had the virulence genotype *plo*/*nanH*/*nanP*/*fimA*/*fimC*/*fimE*, which was the prevalent virulence genotype in our investigation. Nine isolates (10.5%) had the virulence genotype *plo*/*cbpA*/*nanH*/*nanP*/*fimE*, while seven isolates (8.1%) had the virulence genotype *plo*/*cbpA*/*nanH*/*nanP*/*fimA*/*fimC*/*fimE*. Eleven (23.4%) isolates from goats have the genotype *plo*/*nanH*/*nanP*/*fimA*/*fimC*/*fimE*. In isolates taken from goats, it is the predominant pathogenicity genotype. Six (15.4%) sheep isolates had the genotype *plo*/*cbpA*/*nanH*/*nanP*/*fimA*/*fimC*/*fimE*, which is the major virulence genotype in sheep-derived isolates. At least three virulence factors were present in every isolate.

### 3.3. Antimicrobial Susceptibility Testing

Table 4 displays the MIC distribution of antibacterial drugs, including MIC_90_ and MIC_50_ values, as well as susceptibility thresholds. Erythromycin has the broadest distribution of MIC values (0.03~128 μg/mL) in the Macrolides class, while linezolid has a relatively narrow distribution (0.03~0.06) in the Oxazolidinones class. Linezolid, amoxicillin/clavulanic acid, penicillin, cefotaxime, and vancomycin frequently have low MIC_90_ values. Tetracycline, gentamicin, and erythromycin have MIC_90_ values of up to 16 μg/mL.

**Figure 3:**
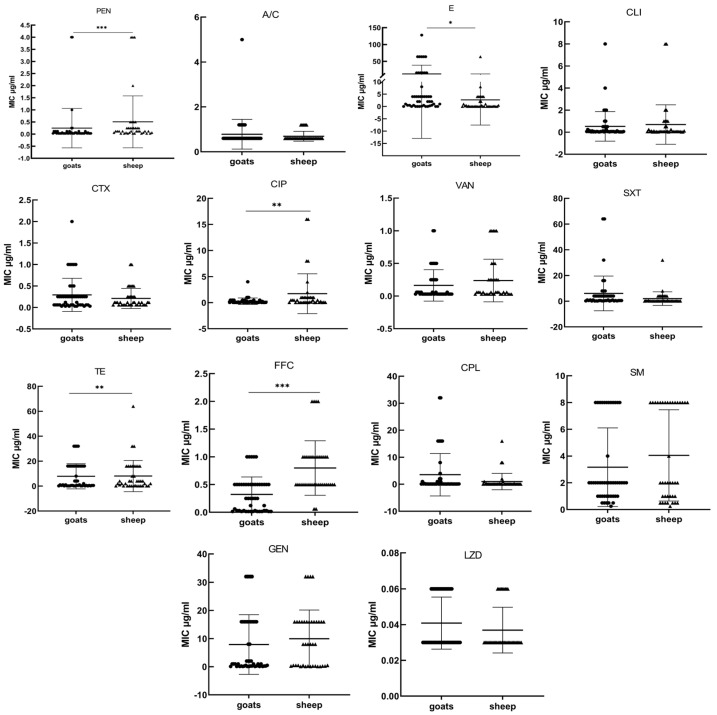
Graphical representation of MIC distribution of 14 antimicrobial agents against *T. pyogenes* isolated from sheep and goats. All the isolates from goats (●), sheep (▲), and origin are represented on the abscissa axis, and every spot represents one single isolate. The results of MIC (μg/mL) of each antimicrobial against the isolates under study are indicated on the ordinate axis. The median is shown with a horizontal black line. * Statistically significant difference among the MIC distribution of *T. pyogenes* populations (* *p* < 0.05, ** *p* < 0.01, *** *p* < 0.001). Abbreviations PEN: Penicillin; A/C: amoxicillin and clavulanate potassium; E: erythromycin; CLI: clindamycin; CTX: cefotaxime; CIP: ciprofloxacin; VAN: vancomycin; SXT: Sulfamethoxazole/Trimethoprim (19/1); TE: tetracycline; FFC: florfenicol; CPL: chloramphenicol; SM: streptomycin; GEN: gentamicin; LZD: linezolid.

As shown in Figure 3, there were significant differences (*p* < 0.05) in the MIC distributions of penicillin, erythromycin, ciprofloxacin, tetracycline, and florfenicol between sheep and goat isolates. There were no statistically significant differences between sheep and goat isolates for the other antibiotics that were examined in this study.

**Figure 4:**
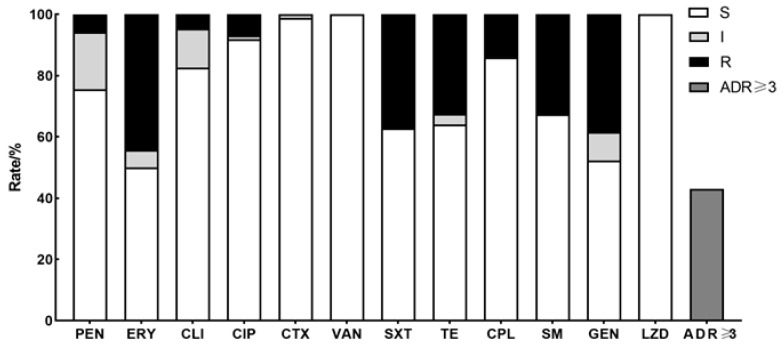
Antimicrobial susceptibility of *T. Pyogenes* isolated from uterine lavage fluid in China. White, green, and grey bars represent the proportion of sensitive strains, moderately resistant strains, and resistant strains, respectively. Dark gray bars represent the respective strain’s proportion of multidrug resistance (MDR ≥ 3).

As shown in Figure 4, an examination of antibacterial medicines with breakpoints revealed that 86 strains of *T. pyogenes* exhibited the highest resistance rate to erythromycin (44.2%, 38/86), followed by gentamicin (38.4%, 33/86) and sulfamethoxazole/trimethoprim (37.2%, 32/86). The resistance rates of *T. pyogenes* isolates to tetracycline and streptomycin were the same (32.6%, 28/86), and to chloramphenicol (14.0%, 12/86), ciprofloxacin (7.0%, 6/86), and penicillin (5.8%, 5/86) and clindamycin (4.7%, 4/86) were resistant to varying degrees. All strains were sensitive to cefotaxime, vancomycin, and linezolid; 37 isolates were resistant to three or more antibiotics and had multidrug resistance (MDR) characteristics. Nine isolates were resistant to four or more antimicrobial agents, two isolates were resistant to five or more antimicrobial agents, and one isolate was resistant to six antimicrobial agents. In addition, 23 strains of bacteria were resistant to two antimicrobial agents, 14 strains were resistant to one antibacterial agent, and 12 strains were sensitive or moderate to all involved antibiotics (Table S2).

### 3.4. Whole Genome Analysis

**Figure 5:**
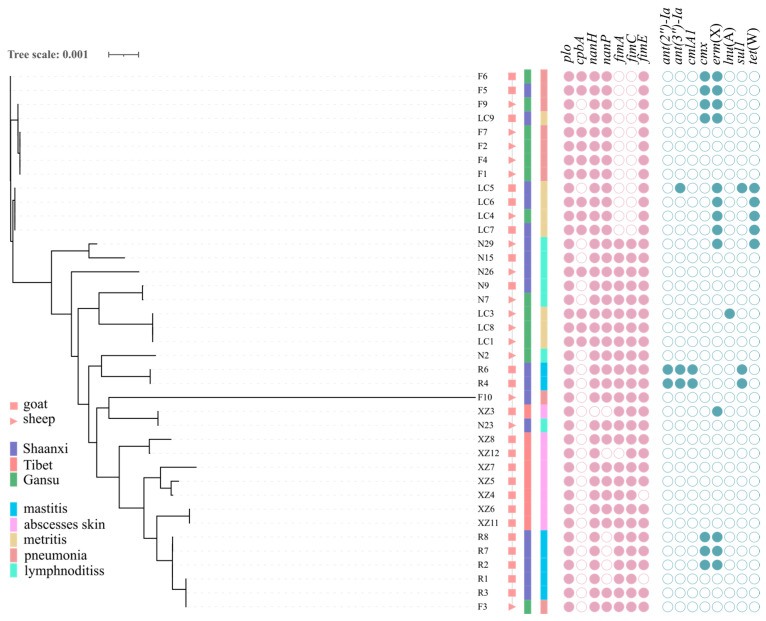
Phylogenetic tree and distribution of virulence genes and antimicrobial resistance genes of the 39 *T. pyogenes* isolates from goats and sheep.

Thirty-nine *T. pyogenes* strains from various clinical lesion samples were chosen for whole-genome sequencing based on their source, virulence genes, and treatment resistance outcomes. Following assembly and analysis, they were uploaded to NCBI. The project number is PRJNA1091699. The genome size and GC ratio are compatible with those found in the GenBank database for *T. pyogenes*. A maximum likelihood phylogenetic tree, constructed using 3556 core-genome SNPs, revealed that *T. pyogenes* isolates typically cluster according to their isolation source, underscoring their close evolutionary ties (Figure 5). Notably, certain isolates from disparate regions, such as F3 from Gansu Province and R3 and R1 from Shaanxi Province, exhibited a close genetic relationship characterized by a minimal number of SNPs (≤10). This finding suggested the possible cross-regional transmission of *T. pyogenes* within China (Figure 5).

Res Finder was used to conduct a comparative analysis of resistance genes on the obtained bacterial genomes. A total of eight resistance genes were detected in 39 isolates, which were *ant(2″)-Ia* and *ant(3″)-Ia* genes that mediate resistance to aminoglycoside antibiotics, *cmlA1* and *cmx* genes that mediate resistance to chloramphenicol antibiotics, an *erm(X)* gene that mediates resistance to macrolide antibiotics, *lnu(A)* that mediates resistance to lincosamide antibiotics, the *sul1* gene that mediates resistance to sulfonamide antibiotics, and the *tet(W)* gene that mediates resistance to tetracycline antibiotics. Except for *erm(X)*, *cmlA*, and *tet(W)*, other drug-resistance genes may be discovered for the first time in *T. pyogenes* isolates from China. Gentamicin resistance is a trait mediated by the *ant(2″)-Ia* and *ant(3″)-Ia* genes. The phenotype mediated by *cmlA1* and *cmx* genes is chloramphenicol resistance; the phenotype mediated by *erm(X)* and *lnu(A)* genes is erythromycin and clindamycin resistance; and the phenotype mediated by *sul1* gene It is resistant to sulfisoxazole, and the *tet(W)* gene-mediated phenotype is resistant to tetracycline, which is consistent with drug susceptibility testing results.

The sequenced and assembled genome sequence was compared to the reference sequence using BLASTN, and eight potential virulence genes were identified. Except for the *fimG* gene, seven known virulence genes were identified. The *plo* gene is present in all sequenced strains, while the *cbpA* gene is detected at 33.3%, the *nanH* gene at 97.4%, the *nanP* gene at 84.6%, and the *fimA*, *fimC*, and *fimE* genes at 66.7%, 69.2%, and 94.9%, respectively. The virulence gene harboring profile of the sequenced bacteria was consistent with the PCR detection findings.

## 4. Discussion

*T. pyogenes* causes mastitis [23] and metritis [24] in dairy cows is widely recognized and reported. In this investigation, we identified *T. pyogenes* from dairy goat and sheep milk samples with mastitis, with isolation rates of 26.1% and 21.0%, respectively. *T. pyogenes* was isolated from birth canal swabs and aborted goat and sheep endometrial tissue at rates of 19.4% and 34.8%, respectively. This is comparable to the isolation rates for postpartum mastitis (14.15%) and metritis (23.83%) in dairy cows [25]. It demonstrates that mastitis and metritis in sheep and goats induced by *T. pyogenes*, which leads to a decline in related production performance, are worthy of consideration. A retrospective investigation in Brazil found that the isolation frequencies of *T. pyogenes* in mastitis, abscess, pneumonia, and subcutaneous abscess were 45.1%, 18.0%, 11.1%, and 9.0%, respectively [26]. Pneumonia and ulcerated skin separated at rates of 23.8% and 37.8%, respectively, in this study. The percentage of sheep pneumonia samples that included isolated *T. pyogenes* was 63.6%. This is consistent with *T. pyogenes*’ high isolation rate in bacterial swine pneumonia in Jilin Province, China [15]. *T. pyogenes* was used to infect and cause bronchoconstriction in porcine PCLS [5]. In addition, the pathological alterations seen during lung sample collection for this investigation demonstrate that *T. pyogenes* infection has a high infection rate and can directly result in lung damage and pneumonia. Subcutaneous abscess in sheep and goats is generally thought to be caused by Corynebacterium pseudotuberculosis [27]. Subcutaneous abscesses are also a clinical manifestation of caseous subcutaneous abscesses. In this survey, *T. pyogenes* was isolated from 27.3% of the samples of subcutaneous abscesses. In addition, we also isolated C. pseudotuberculosis and Staphylococcus aureus, suggesting that *T. pyogenes* may be involved in these infections in conjunction with other bacteria. However, further research is needed to determine the specific role of *T. pyogenes* in caseous lymphadenitis.

Previous studies have revealed that the pathogenicity of bacteria is strongly connected to the virulence genes they contain [28]. There are now eight identified potential virulence genes in *T. pyogenes*, with *plo* being detectable in all isolates [29]; the carrying rate of the *plo* gene in the present study was 100%, which was totally consistent with prior findings. This demonstrates that PLO is critical to the survival and infection of *T. pyogenes*. At the same time, this gene is a critical target for clinical trials and vaccine development. Genes encoding fimbriae are associated with bacterial adherence to host cells during *T. pyogenes* infection. In this study, the carrying rates of *fimA*, *fimC*, and *fimE* were 75.6%, 66.3%, and 66.3%, respectively, which is broadly consistent with the findings of several investigations on pilus gene distributions [30,31]. The *fimA* gene is widely thought to be the main pilus in *T. pyogenes* [30]. In this study, the carrying rate of the *fimA* gene was the highest among all known genes encoding fimbriae, which confirmed this conclusion from one aspect. *cbpA* is a collagen-binding protein that promotes epithelial cell and fibroblast adhesion during *T. pyogenes* infection [32]. The isolation rate of *cbpA* in this study is 54.7%, demonstrating that while the *cbpA* gene appears in isolates from a variety of sources, its isolation frequency has varied thus far [14,33,34], It lacks obvious characteristics, and its expression regulation in the bacterial invasion process deserves further investigation. Schaufuss and Lämmler were the first to report on *T. pyogenes* neuraminidase 50 kDa annotation [35]. Later, two neuraminidase enzymes expressed by *T. pyogenes* were better characterized [36,37]. According to research, neuraminidase NanH and NanP are vital in host tissue colonization because they cleave host cell terminal sialic acid residues and reduce tissue mucus viscosity [38]. In this investigation, *nanH* and *nanP* genes were found in 80.2% and 59.3% of isolates, respectively. This result is consistent with prior results for *T. pyogenes* isolates from cattle [34,39,40].

Bacterial resistance has become a major danger to worldwide public health. The World Health Organization considers overuse of antibiotics, incomplete treatment duration, inappropriate selection of antimicrobial agents, and the transfer of antibiotic resistance genes (ARGs) between bacteria to be the primary causes of increasing bacterial antimicrobial resistance [41]. The 86 strains of *T. pyogenes* isolated in this study were resistant to macrolide erythromycin (44.2%, 38/86), sulfa drugs sulfisoxazole (37.2%, 32/86), and Amoxicillin/Clavulanic acid (38.4%,33/86), streptomycin (32.6%, 28/86), tetracycline (32.6%, 28/86), chloramphenicol (14.0%, 12/86), β-lactams penicillin (5.8%, 5/86) and lincosamide clindamycin (4.7%, 4/86) had varying degrees of resistance. At the same time, the drug resistance phenotypes and genotypes of the 39 isolates investigated via whole genome sequencing were identical. The most significant resistance genes revealed in recent investigations are *tet(W)*, which mediates tetracycline antibiotic resistance, and *erm(X)*, which mediates macrolide antibiotic resistance [42]. The *ant(2″)-Ia* and *ant(3″)-Ia* genes mediate resistance to aminoglycoside antibiotics, The *cmlA1* and *cmx* genes mediate chloramphenicol antibiotic resistance, the *lnu(A)* genes mediate macrolide antibiotics, and the sulfonamide-mediated antibiotic resistance *sul1* gene may be discovered for the first time in *T. pyogenes* isolates from sheep and goats in China. At the same time, the fraction of multi-drug-resistant strains in this study reached 43%, indicating that *T. pyogenes* resistance is constantly increasing and there is a risk of drug-resistant gene transfer. Farm veterinarians and associated researchers should pay close attention.

## 5. Conclusions

In summary, 86 strains of *T. pyogenes* were isolated from various clinical lesion tissues of goats and sheep in western China. Their virulence genes, drug resistance genes, and genetic evolutionary relationships were identified and studied. A total of 43.0% of the isolates exhibited multidrug resistance and carried a large number of drug-resistant genes. We posit that *T. pyogenes* may pose a significant challenge to the sheep and goat farming industry in western China. The inability to effectively and safely prevent and treat multidrug-resistant *T. pyogenes* will present major difficulties and threats to food safety, animal husbandry, and even human health. This study recommends exercising caution when using antibiotics to treat related diseases caused by *T. pyogenes* in goat and sheep pastures. Long-term monitoring of antibiotic resistance and pathogenicity of *T. pyogenes* is recommended.

## Figures and Tables

**Table 1:** Sample statistics of different clinical manifestations.

Clinical Manifestation Specie	Specie		Total
	Goat	Sheep	
Mastitis	38	19	57
Pneumonia	31	11	42
Subcutaneous abscess	67	54	121
Metritis	36	23	59
Ulcerated skin	37	0	37
Total	209	107	316

**Table 2:** Analysis of *T. pyogenes* isolates from sheep and goats with various clinical manifestations.

Clinical Manifestation Specie	Mastitis	Pneumonia	Subcutaneous Abscess	Metritis	Ulcerated Skin	Total of Isolates (%)
	n Isolates/T Isolates (%)	n Isolates/T Isolates (%)	n Isolates/T Isolates (%)	n Isolates/T Isolates (%)	n Isolates/T Isolates (%)	
Goat	26.1 (10/38)	9.7 (3/31)	28.4 (19/67)	19.4 (7/36)	37.8 (14/37)	22.5 (47/209)
Sheep	21.0 (4/19)	63.6 (7/11)	25.9 (14/54)	34.8 (8/23)	—	36.4 (39/107)
Total	24.6 (14/57)	23.8 (10/42)	27.3 (33/121)	25.4 (15/59)	37.8 (14/37)	27.2 (86/316)

**Table 3:** Prevalence of virulence genes of *T. pyogenes* isolated from different clinical manifestations in sheep and goats in western China (n = 86).

Virulence Gene	N (%) of *T. pyogenes* Strains Isolated From				
	Pneumonia	Subcutaneous Abscess	Metritis	Mastitis	Ulcerated Skin
*plo*	10(100)	33(100)	14(100)	15(100)	14(100)
*cbpA*	7(70.0)	19(57.6)	6(42.9)	11(73.3)	4(28.6)
*nanH*	8(80.0)	21(63.6)	12(85.7)	15(100)	13(92.9)
*nanP*	8(80.0)	16(48.5)	6(42.9)	13(86.7)	8(57.1)
*fimA*	3(30.0) ^b^	27(81.8) ^a^	12(85.7) ^a,b^	10(66.7) ^a,b^	13(92.9) ^a^
*fimC*	3(30.0))	25(75.8)	10(71.4))	7(46.7)	12(85.7)
*fimE*	9(90.0))	19(57.6)	11(78.6)	11(73.3)	7(50.0)

The letters indicate the level of significant difference between groups (*p* < 0.05).

**Table 4:** Minimum Inhibitory Concentration (MIC) distributions, MIC50 and MIC90 of 14 antimicrobial agents against 86 *Trueperella pyogenes* isolates from sheep and goats.

Antimicrobial	No. of Isolates at Indicated Antimicrobial Dilution (μg/mL)																	MIC_50_ (μg/mL)	MIC_90_ (μg/mL)
	≤0.015	0.03	0.06	0.12	0.25	0.5	1	2	4	8	16	32	64	128	256	512	1024		
PEN ^1^	0	36	12	17	10	4	1	1	5	0	0	0	0	0	0	0	0	0.06	0.5
A/C ^1^	0	0	72	13	1	0	0	0	0	0	0	0	0	0	0	0	0	0.06/0.03	0.12
E ^2^	0	26	0	6	3	8	5	7	16	2	6	0	6	1	0	0	0	0.5	16
CLI ^3^	0	19	33	8	4	7	6	5	1	3	0	0	0	0	0	0	0	0.06	2
CIP ^4^	0	26	13	9	6	13	12	1	2	2	2	0	0	0	0	0	0	0.12	1
CTX ^1^	0	7	28	13	22	8	7	1	0	0	0	0	0	0	0	0	0	0.12	0.5
VAN ^5^	0	40	17	0	13	9	7	0	0	0	0	0	0	0	0	0	0	0.06	0.5
SXT ^6^	0	0	0	18	17	14	5	0	22	4	2	2	2	0	0	0	0	0.5/9.5	8/152
TE ^7^	0	0	0	21	3	10	5	6	10	3	21	6	1	0	0	0	0	2	16
FFC ^8^	11	6	3	2	6	36	18	4	0	0	0	0	0	0	0	0	0	0.5	1
CPL ^8^	0	0	46	16	4	0	5	3	1	3	6	2	0	0	0	0	0	0.06	8
SM ^9^	0	0	0	0	2	12	16	26	2	28	0	0	0	0	0	0	0	2	8
GEN ^9^	0	0	0	14	13	7	7	4	0	8	24	9	0	0	0	0	0	2	16
LZD ^10^	0	60	26	0	0	0	0	0	0	0	0	0	0	0	0	0	0	0.03	0.06

PEN: Penicillin; A/C: Amoxicillin/Clavulanic acid (2/1); E: Erythromycin; CLI: Clindamycin; CIP: Ciprofloxacin; CTX: Cefotaxime; VAN: Vancomycin; SXT: Sulfamethoxazole/Trimethoprim (19/1); TE: Tetracycline; FFC: Florfenicol; CPL: Chloramphenicol; SM: Streptomycin; GEN: Gentamicin; LZD: Linezolid; ^1^ β-lactams; ^2^ Macrolides; ^3^ Lincosamides; ^4^ Fluoroquinolone; ^5^ Glycopeptides; ^6^ Sulphonamide; ^7^ Tetracycline; ^8^ Chloramphenicols; ^9^ Aminoglycosides; ^10^ Oxazolidinones. The breakpoints indicated in CLSI M45 are represented by black vertical lines, and the breakpoints described in the sources that are referenced in 2.5 are represented by blue vertical lines. Amoxicillin/Clavulanic acid and Florfenicol: No breakpoints were identified with florfenicol and amoxicillin/clavulanic acid.

## Data Availability

The authors confirm that all data is fully available without restriction. The raw sequencing reads were deposited into the NCBI Sequence Read Archive (SRA) database (Accession Number: PRJNA1091699).

## Supplementary Materials

The following supporting information can be downloaded at https://www.mdpi.com/article/10.3390/ani14202964/s1, Table S1: Virulotypes of *T. pyogenes* isolates from goats and sheep; Table S2. Statistics on antibiotic resistance among 86 strains of *T. pyogenes*.
