# Supplementary material for: Prevalence, Virulence Genes, Drug Resistance and Genetic Evolution of Trueperella pyogenes in Small Ruminants in Western China
Source: Animals (Basel). 2024 Oct 14;14(20):2964. doi: 10.3390/ani14202964 (PMC11503795; doi:10.3390/ani14202964)
Supplement: Supplementary file 1 [file animals-14-02964-s001.zip › supplementary files/Table S2.pdf]

Table S2. Statistics on antibiotic resistance among 86 strains of T.pyogenes.

	PEN	ERY	CLI	CIP	CTX	VAN	SXT	TE	CPL	SM	GEN	LZD	Number of resistant antibiotics.
F1	S	S	S	S	S	S	S	S	S	S	S	S	0
F2	I	I	S	S	S	S	S	S	S	S	S	S	0
F3	I	I	I	R	S	S	S	S	S	R	S	S	2
F4	I	R	S	S	S	S	S	S	S	R	S	S	2
F5	S	R	S	S	S	S	R	S	R	S	S	S	3
F6	I	R	S	S	S	S	S	S	R	S	S	S	2
F7	I	R	S	R	S	S	S	S	S	S	R	S	3
F8	I	R	S	R	I	S	S	R	R	S	S	S	4
F9	S	R	R	I	S	S	S	S	R	R	R	S	5
F10	S	S	I	S	S	S	S	S	S	R	S	S	1
LC1	I	S	I	S	S	S	S	R	S	S	S	S	1
LC2	S	S	S	S	S	S	S	R	S	R	R	S	3
LC3	S	S	R	S	S	S	S	R	S	S	R	S	3
LC4	I	R	I	S	S	S	R	R	S	S	S	S	3
LC5	S	R	S	S	S	S	R	R	S	S	S	S	3
LC6	S	R	S	S	S	S	S	R	S	S	S	S	2
LC7	S	R	S	S	S	S	S	R	S	S	S	S	2
LC8	I	S	S	S	S	S	R	S	S	S	S	S	1
LC9	S	R	I	S	S	S	R	R	R	S	S	S	4
LC10	S	S	S	R	S	S	S	R	S	S	I	S	2
LC11	S	R	S	S	S	S	S	S	S	R	R	S	3
LC12	S	R	S	S	S	S	R	S	S	S	I	S	2

LC13	R	R	S	S	S	S	R	R	S	S	S	S	4
LC14	R	R	S	S	S	S	R	S	S	S	R	S	4
LC15	R	S	S	S	S	S	S	S	S	R	R	S	3
N1	I	S	S	S	S	S	S	S	S	S	S	S	0
N2	S	S	S	S	S	S	S	S	S	S	S	S	0
N3	S	R	S	S	S	S	R	S	S	S	R	S	3
N4	S	R	S	S	S	S	R	S	S	S	R	S	3
N5	S	R	S	S	S	S	R	S	S	S	S	S	2
N6	I	S	S	S	S	S	S	S	S	S	R	S	1
N7	I	S	S	S	S	S	S	S	S	S	S	S	0
N8	I	S	S	S	S	S	S	S	S	S	I	S	0
N9	S	R	S	S	S	S	S	R	S	S	S	S	2
N10	S	S	S	S	S	S	S	I	S	S	S	S	0
N11	I	S	S	S	S	S	S	S	S	R	R	S	2
N12	S	R	S	S	S	S	R	S	S	R	R	S	4
N13	S	S	S	S	S	S	S	R	S	R	R	S	3
N14	I	S	S	S	S	S	S	R	S	R	R	S	3
N15	S	S	S	S	S	S	S	R	S	S	R	S	2
N16	S	S	S	R	S	S	S	S	S	S	R	S	2
N17	S	S	S	S	S	S	R	R	S	R	S	S	3
N18	R	S	S	S	S	S	S	R	S	S	I	S	2
N19	R	S	S	S	S	S	S	R	S	S	S	S	2
N20	S	S	S	S	S	S	S	R	S	S	I	S	1
N21	I	S	S	S	S	S	S	R	S	S	R	S	2
N22	S	R	S	S	S	S	S	R	S	R	R	S	4
N23	S	R	S	S	S	S	S	R	S	S	S	S	2

**N24**	**S**	**R**	**S**	**S**	**S**	**S**	**S**	**R**	**S**	**R**	**R**	**S**	**4**
**N25**	**S**	**R**	**I**	**S**	**S**	**S**	**S**	**R**	**S**	**R**	**R**	**S**	**4**
**N26**	**S**	**R**	**I**	**S**	**S**	**S**	**R**	**S**	**S**	**S**	**S**	**S**	**2**
**N27**	**S**	**S**	**S**	**S**	**S**	**S**	**S**	**I**	**S**	**R**	**I**	**S**	**1**
**N28**	**S**	**S**	**S**	**R**	**S**	**S**	**S**	**S**	**S**	**R**	**S**	**S**	**2**
**N29**	**S**	**R**	**R**	**S**	**S**	**S**	**R**	**R**	**R**	**S**	**S**	**S**	**5**
**N30**	**S**	**R**	**S**	**S**	**S**	**S**	**R**	**I**	**S**	**R**	**I**	**S**	**3**
**N31**	**S**	**R**	**S**	**S**	**S**	**S**	**R**	**S**	**S**	**R**	**R**	**S**	**4**
**N32**	**S**	**R**	**S**	**S**	**S**	**S**	**S**	**S**	**S**	**R**	**R**	**S**	**3**
**N33**	**S**	**R**	**S**	**S**	**S**	**S**	**S**	**S**	**S**	**R**	**R**	**S**	**3**
**R1**	**S**	**S**	**S**	**S**	**S**	**S**	**S**	**S**	**S**	**S**	**R**	**S**	**1**
**R2**	**S**	**R**	**S**	**S**	**S**	**S**	**S**	**S**	**R**	**S**	**R**	**S**	**3**
**R3**	**S**	**R**	**I**	**S**	**S**	**S**	**S**	**S**	**S**	**S**	**S**	**S**	**1**
**R4**	**S**	**R**	**S**	**S**	**S**	**S**	**R**	**S**	**S**	**S**	**R**	**S**	**3**
**R5**	**S**	**S**	**I**	**S**	**S**	**S**	**R**	**S**	**R**	**S**	**R**	**S**	**3**
**R6**	**S**	**S**	**S**	**S**	**S**	**S**	**R**	**S**	**R**	**S**	**R**	**S**	**3**
**R7**	**S**	**R**	**I**	**S**	**S**	**S**	**S**	**S**	**R**	**S**	**S**	**S**	**2**
**R8**	**S**	**R**	**I**	**S**	**S**	**S**	**S**	**S**	**R**	**S**	**S**	**S**	**2**
**R9**	**S**	**S**	**S**	**S**	**S**	**S**	**R**	**S**	**S**	**R**	**R**	**S**	**3**
**R10**	**S**	**S**	**S**	**S**	**S**	**S**	**R**	**S**	**S**	**R**	**R**	**S**	**3**
**R11**	**S**	**R**	**S**	**S**	**S**	**S**	**R**	**R**	**R**	**R**	**R**	**S**	**6**
**R12**	**S**	**S**	**S**	**S**	**S**	**S**	**R**	**S**	**S**	**R**	**R**	**S**	**3**
**R13**	**S**	**S**	**S**	**S**	**S**	**S**	**R**	**S**	**S**	**S**	**R**	**S**	**3**
**R14**	**S**	**S**	**S**	**S**	**S**	**S**	**R**	**S**	**S**	**R**	**I**	**S**	**3**
**XZ1**	**S**	**R**	**S**	**S**	**S**	**S**	**S**	**S**	**S**	**S**	**S**	**S**	**1**
**XZ2**	**S**	**S**	**S**	**S**	**S**	**S**	**S**	**S**	**S**	**S**	**S**	**S**	**0**

**XZ3**	**S**	**R**	**R**	**S**	**S**	**S**	**S**	**S**	**S**	**S**	**S**	**S**	**2**
**XZ4**	**S**	**S**	**S**	**S**	**S**	**S**	**S**	**S**	**S**	**R**	**S**	**S**	**0**
**XZ5**	**S**	**S**	**S**	**S**	**S**	**S**	**S**	**S**	**S**	**S**	**S**	**S**	**0**
**XZ6**	**S**	**I**	**S**	**S**	**S**	**S**	**S**	**S**	**S**	**R**	**S**	**S**	**1**
**XZ7**	**S**	**I**	**S**	**S**	**S**	**S**	**S**	**S**	**S**	**S**	**S**	**S**	**0**
**XZ8**	**S**	**I**	**S**	**S**	**S**	**S**	**S**	**S**	**S**	**S**	**S**	**S**	**0**
**XZ9**	**S**	**S**	**S**	**S**	**S**	**S**	**R**	**S**	**S**	**S**	**S**	**S**	**1**
**XZ10**	**S**	**S**	**S**	**S**	**S**	**S**	**R**	**S**	**S**	**S**	**S**	**S**	**1**
**XZ11**	**S**	**S**	**S**	**S**	**S**	**S**	**R**	**R**	**S**	**S**	**S**	**S**	**2**
**XZ12**	**S**	**S**	**S**	**S**	**S**	**S**	**R**	**R**	**S**	**S**	**S**	**S**	**2**
**XZ13**	**S**	**S**	**S**	**S**	**S**	**S**	**R**	**S**	**S**	**S**	**S**	**S**	**1**
**XZ14**	**S**	**S**	**S**	**S**	**S**	**S**	**R**	**S**	**S**	**S**	**S**	**S**	**1**
