# Supplementary material for: Prevalence, Virulence Genes, Drug Resistance and Genetic Evolution of Trueperella pyogenes in Small Ruminants in Western China
Source: Animals (Basel). 2024 Oct 14;14(20):2964. doi: 10.3390/ani14202964 (PMC11503795; doi:10.3390/ani14202964)
Supplement: Supplementary file 1 [file animals-14-02964-s001.zip › supplementary files/Table S1.pdf]

Table S1 Virulotypes of T. pyogenes isolates from goats and sheep

Genotype							Total No. of isolates	%	Goats No. of isolates	%	Sheep No. of isolates	%
plo	cbpA	nanH	nanP	fimA	fimC	fimE	7	8.1	1	2.1	6	15.4
plo	cbpA	nanH	nanP	fimA	fimC		4	4.7	4	8.5	0	0
plo	cbpA	nanH	nanP	fimA	fimE		1	1.2	0	0	1	2.6
plo	cbpA	nanH	nanP	fimA			2	2.3	2	4.2	0	0
plo	cbpA	nanH	nanP	fimE			9	10.5	4	8.5	5	12.8
plo	cbpA	nanH	nanP				3	3.5	3	6.4	0	0
plo	cbpA	nanH	fimA	fimC			5	5.8	4	8.5	1	2.6
plo	cbpA	nanH	fimA	fimE			3	3.5	0	0	3	7.7
plo	cbpA	nanH	fimA				2	2.3	1	2.1	1	2.6
plo	cbpA	nanP	fimE				1	1.2	0	0	1	2.6
plo	cbpA	nanP					1	1.2	0	0	1	2.6
plo	cbpA	fimA	fimC	fimE			3	3.5	1	2.1	2	5.1
plo	cbpA	fimC	fimE				2	2.3	0	0	2	5.1
plo	cbpA	fimA	fimC				1	1.2	0	0	1	2.6
plo	cbpA	fimA					2	2.3	0	0	2	5.1
plo	nanH	nanP	fimA	fimC	fimE		16	18.6	11	23.4	5	12.8
plo	nanH	nanP	fimA	fimC			5	5.8	5	10.6	0	0
plo	nanH	nanP	fimC	fimE			1	1.2	1	2.1	0	0
plo	nanH	nanP	fimE				3	3.5	2	4.2	1	2.6
plo	nanH	fimA	fimC	fimE			3	3.5	3	6.4	0	0
plo	nanH	fimA	fimC				2	2.3	2	4.2	0	0
plo	nanH	fimC	fimE				2	2.3	1	2.1	1	2.6
plo	nanH	fimC					1	1.2	0	0	1	2.6
plo	cbpA	fimA					1	1.2	1	2.1	0	0
plo	fimA	fimC	fimE				5	5.8	1	2.1	4	10.2
plo	fimA	fimE					1	1.2	0	0	1	2.6
